# Supplementary material for: Step-by-Step Immune Activation for Suicide Gene Therapy Reinforcement
Source: Int J Mol Sci. 2021 Aug 29;22(17):9376. doi: 10.3390/ijms22179376 (PMC8430744; doi:10.3390/ijms22179376)
Supplement: Supplementary file 1 [file ijms-22-09376-s001.zip › ijms-1344095-supplementary.pdf]

Table S1: Clinical trials of HSVtk/prodrug and CD/5-FC systems

Trial No.	Start-Completion	Phase	Patients	Vector Used	Suicide gene/Prodrug	Combination	Result	Sponsor
**HCVtk/prodrug**								
NCT01997190	2013-2020	I	Malignant pleural effusion, Lung cancer, Mesothelioma, Breast cancer, Ovarian cancer	AdV	HSVtk/valACV	Standard chemotherapy* + Celecoxib (before or after AdVtk administration)	Completed	Candel Therapeutics, Inc.
NCT00634231	2008-2021	I	Malignant glioma, Recurrent ependymoma	AdV	HSVtk/valACV	RT + standard chemotherapy*	Ongoing	Candel Therapeutics, Inc.
NCT03576612	2018-2021	I	Malignant glioma,	AdV	HSVtk/valACV	RT + Temozolomide + Nivolumab	Ongoing	Sidney Kimmel Comprehensive Cancer Center at Johns Hopkins
NCT01811992	2013-2021	I	Malignant glioma, Glioblastoma multiforme	AdV	HSVtk/valACV	Flt3L + RT + chemotherapy	Ongoing	University of Michigan Rogel Cancer Center
NCT00964756	2009-2013	I	Ovarian cancer	AdV	HSVtk/GCV	N/A	Completed	University of Alabama at Birmingham
NCT00844623	2002-2008	I	Hepatocellular carcinoma	Defective adenovirus	HSVtk/valACV/GCV	N/A	Completed	Clinica Universidad de Navarra, Universidad de Navarra
NCT00638612	2008-2015	I	Pancreatic adenocarcinoma, Pancreatic cancer	AdV	HSVtk/valACV	Surgery and/or chemoradiation	Completed	Advantagene, Inc.
NCT00751270	2005-2011	I	Malignant glioma, Glioblastoma multiforme, Anaplastic astrocytoma	AdV	HSVtk/valACV	RT + standard chemotherapy	Completed	Advantagene, Inc.
NCT00005057	2000-N/A	I	Melanoma	AdV	HSVtk/GCV	N/A	Completed	National Human Genome Research Institute (NHGRI)
NCT03338777	2020-2020	I	Melanoma	Lipoplexes	HSVtk/GCV	IL2, GM-CSF	Terminated (Failure to achieve primary objective)	Hospital Italiano de Buenos Aires

NCT03131037	2017-2022	I	Lung Cancer	AdV	HSVtk/valACV	N/A	Ongoing	Candel Therapeutics, Inc., University of Pennsylvania
NCT00002824	1996-N/A	I	Brain and Central Nervous System Tumors	AdV	HSVtk/GCV	N/A	Completed	Abramson Cancer Center of the University of Pennsylvania
NCT04313868	2014-2022	I	Hepatocellular Carcinoma, Metastatic Cancer	Unknown	HSVtk/valACV	GM-CSF	Ongoing	GenVivo, Inc.
NCT03596086	2017-2025	I, II	Glioblastoma Multiforme, Astrocytoma	AdV	HSVtk/valACV	RT + standard chemotherapy	Ongoing	The Methodist Hospital Research Institute
NCT03603405	2018-2025	I, II	Glioblastoma, Anaplastic Astrocytoma	AdV	HSVtk/valACV	RT + standard chemotherapy	Ongoing	The Methodist Hospital Research Institute
NCT01913106	2007-2028	I, II	Prostate cancer	AdV	HSVtk/valACV	RT (brachytherapy)	Ongoing	The Methodist Hospital System
NCT02768363	2016-2021	II	Prostate cancer	AdV	HSVtk/valACV	N/A	Ongoing	Candel Therapeutics, Inc.
NCT02202564	2014-2014	II	Liver cancer, Hepatocellular carcinoma	AdV	HSVtk/GCV	Liver transplantation	Completed	Huazhong University of Science and Technology
NCT04495153	2020-2021	II	Non small cell lung cancer	AdV	HSVtk/valACV	ICI + standard chemotherapy + RT	Ongoing	Candel Therapeutics, Inc.
NCT02446093	2015-2020	II	Pancreatic adenocarcinoma	AdV	HSVtk/valACV	Standard chemotherapy + RT + surgery	Ongoing	Candel Therapeutics, Inc.
NCT03004183	2017-2023	II	Metastatic non-small cell lung cancer, Metastatic triple-negative breast cancer	AdV	HSVtk/valACV	SBRT + ICI	Ongoing	Jenny C. Chang, MD, Merck Sharp & Dohme Corp.
NCT02831933	2017-2020	II	Lung squamous cell carcinoma, Nonsquamous nonsmall cell neoplasm of lung, Metastatic uveal melanoma	AdV	HSVtk/valACV	SBRT + ICI	Terminated (Lack of funding)	Eric Bernicker, MD

NCT03541928	2018-2023	II	Prostate cancer	AdV	HSVtk/valACV	Androgen deprivation therapy + Brachytherapy + External beam radiotherapy + Prostatectomy	Ongoing	The Methodist Hospital System
NCT00589875	2007-2016	II	Malignant Glioma, Glioblastoma Multiforme, Anaplastic Astrocytoma	AdV	HSVtk/valACV	RT + standard chemotherapy	Completed	Advantagene, Inc.
NCT00870181	2008-2012	II	Malignant glioma of brain, Glioblastoma	AdV	HSVtk/valACV	N/A	Completed	Huazhong University of Science and Technology
NCT01436968	2011-2021	III	Prostate cancer	AdV	HSVtk/valACV	RT	Ongoing	Candel Therapeutics, Inc.
**CD/5-FC**								
NCT01470794	2012-2016	I	Glioblastoma multiforme, Anaplastic astrocytoma, Anaplastic oligodendroglioma, Anaplastic oligoastrocytoma	RRV	CD/5-FC	N/A	Completed	Tocagen Inc.
NCT01985256	2014-2016	I	Glioblastoma multiforme, Anaplastic astrocytoma, Anaplastic oligodendroglioma, Anaplastic oligoastrocytoma	RRV	CD/5-FC	N/A	Completed	Tocagen Inc.
NCT04327011	2011-2019	I	Glioblastoma multiforme, Astrocytoma, Malignant gliomas	RRV	CD/5-FC	N/A	Terminated (Sponsor decision)	Tocagen Inc.
NCT01156584	2010-2016	I	Glioblastoma multiforme, Anaplastic astrocytoma, Anaplastic oligodendroglioma,	RRV	CD/5-FC	N/A	Completed	Tocagen Inc.

			Anaplastic oligoastrocytoma					
NCT02598011	2017-2022	I	Newly diagnosed high grade glioma (HGG)	RRV	CD/5-FC	N/A	Withdrawn (Sponsor decision)	Tocagen Inc.
NCT04195373	2019-2020	I	Gastrointestinal cancer	OV	CD/5-FC	ICI	Withdrawn	Themis Bioscience GmbH
NCT04089163	2019-2020	I	Bladder cancer	RRV	CD/5-FC	N/A	Withdrawn (Sponsor decision)	Tocagen Inc.
NCT00978107	2009-2014	I	Hepatocellular carcinoma	MVA	CD/5-FC	N/A	Completed	Transgene
NCT02576665	2016-2019	I	Colorectal cancer, Triple negative breast cancer, Pancreatic cancer, Non-small cell lung cancer, Head and neck cancer, Ovarian cancer, Lymphoma, Sarcoma, Bladder cancer, Melanoma	RRV	CD/5-FC	N/A	Terminated (Sponsor decision)	Tocagen Inc.
NCT04194034	2019-2021	I, II	Colorectal neoplasm	OV	CD/5-FC	N/A	Ongoing	Transgene
NCT04226066	2020-2020	I, II	Advanced malignant solid tumors	OV	CD/5-FC	N/A	Ongoing	Tasly Tianjin Biopharmaceutical Co., Ltd.
NCT03294486	2017-2017	I, II	Glioblastoma, Brain cancer	OV	CD/5-FC	N/A	Ongoing	Assistance Publique - Hôpitaux de Paris
NCT03724071	2018-2021	I, II	Colorectal neoplasm, Digestive system neoplasm	OV	CD/5-FC	N/A	Ongoing	Transgene
NCT02414165	2015-2019	II, III	Glioblastoma multiforme, Anaplastic astrocytoma	RRV	CD/5-FC	N/A	Terminated (Sponsor Decision)	Tocagen Inc.
**CD/5-FC +HSVtk/vGCV**								
NCT03281382	2017-2021	I	Metastatic pancreatic cancer	Oncolytic AdV	CD/5-FC +HSVtk/vGCV	IL12	Unknown	Henry Ford Health System
NCT03029871	2017-2022	I	Non-small cell lung cancer stage I	Oncolytic AdV	CD/5-FC +HSVtk/vGCV	SBRT	Withdrawn (No	Benjamin Movsas, M.D.

							participants were enrolled)	
NCT04739046	2021-2024	II	Pancreas Cancer	AdV	CD/5-FC +HSVtk/vGCV	RT	Ongoing	Seoul National University Bundang Hospital, NewGenPharm Incoporation
NCT00583492	2007-2013	II	Prostate Cancer	AdV	CD/5-FC +HSVtk/vGCV	IMRT	Completed	Henry Ford Health System

HSVtk – herpes simplex virus thymidine kinase gene
CD - cytosine deaminase gene
ValACV – valacyclovir, Valtrex
GCV - gancyclovir
RT - radiotherapy
AdV - adenovirus
OV – oncolytic virus
RRV – retroviral replicating vector
MVA - vaccinia virus Ankara
HSV1 - Herpes simplex virus 1
5-FC – 5-flucytosine, Ancotil
ICI - immune checkpoint inhibitor
SBRT - stereotactic body radiation therapy
IMRT - intensity modulated radiotherapy
* - allowed after prodrug administration
N/A – not applicable
